# Indirect p53-dependent transcriptional repression of *Survivin, CDC25C,* and *PLK1* genes requires the cyclin-dependent kinase inhibitor p21/CDKN1A and CDE/CHR promoter sites binding the DREAM complex

**DOI:** 10.18632/oncotarget.6356

**Published:** 2015-11-22

**Authors:** Martin Fischer, Marianne Quaas, Annina Nickel, Kurt Engeland

**Affiliations:** ^1^ Molecular Oncology, Medical School, University of Leipzig, Leipzig, Germany; ^2^ Department of Medical Oncology, Dana–Farber Cancer Institute, Department of Medicine, Brigham and Women's Hospital, Harvard Medical School, Boston, MA, USA

**Keywords:** p53, CDKN1A, DREAM, CDE-CHR, p53-p21-DREAM-CDE/CHR pathway

## Abstract

The transcription factor p53 is central to cell cycle control by downregulation of cell cycle-promoting genes upon cell stress such as DNA damage. *Survivin* (*BIRC5*), *CDC25C*, and *PLK1* encode important cell cycle regulators that are repressed following p53 activation. Here, we provide evidence that p53-dependent repression of these genes requires activation of *p21* (*CDKN1A, WAF1, CIP1*). Chromatin immunoprecipitation (ChIP) data indicate that promoter binding of B-MYB switches to binding of E2F4 and p130 resulting in a replacement of the MMB (Myb-MuvB) by the DREAM complex. We demonstrate that this replacement depends on p21. Furthermore, transcriptional repression by p53 requires intact DREAM binding sites in the target promoters. The CDE and CHR cell cycle promoter elements are the sites for DREAM binding. These elements as well as the p53 response of *Survivin*, *CDC25C*, and *PLK1* are evolutionarily conserved. No binding of p53 to these genes is detected by ChIP and mutation of proposed p53 binding sites does not alter the p53 response. Thus, a mechanism for direct p53-dependent transcriptional repression is not supported by the data. In contrast, repression by DREAM is consistent with most previous findings and unifies models based on p21-, E2F4-, p130-, and CDE/CHR-dependent repression by p53. In conclusion, the presented data suggest that the p53-p21-DREAM-CDE/CHR pathway regulates p53-dependent repression of *Survivin*, *CDC25C*, and *PLK1.*

## INTRODUCTION

Progression through the cell cycle is a tightly regulated process. Cell cycle control involves mechanisms such as protein phosphorylation and dephosphorylation, transcriptional control, proteolysis, and protein complex formation. Survivin (BIRC5), CDC25C phosphatase, and Polo-like kinase 1 (PLK1) are central regulators of the cell cycle.

Survivin forms the chromosomal passenger complex (CPC) together with Aurora B, Borealin (CDCA8), and INCENP [1]. As a member of the CPC, survivin plays an important role in regulating chromosome-microtubule attachment, the spindle assembly checkpoint, and cytokinesis [1]. Survivin exerts a strong anti-apoptotic behavior, is overexpressed in many tumor types, and is a target for anti-tumor therapy [1-3].

The phosphatase CDC25C dephosphorylates the cyclin-dependent kinase CDK1/CDC2, thereby activating Cyclin B/CDK1 kinase complex, which is a key step for cell cycle progression into mitosis [4]. Microinjection of purified CDC25C protein drives cells into mitosis [5]. Furthermore, CDC25C overexpression in tumor tissues has been observed which underscores an oncogenic function of CDC25C [6-9]. Consequently, CDC25C has also been a target for therapeutic intervention [10-12].

PLK1 is a member of the Polo-like kinase family with five paralogs in vertebrates [13-15]. After recognition of substrates by two C-terminal non-catalytic phospho-serine/threonine binding domains, the Polo-box domains (PBD), PLK1 is able to phosphorylate serines and threonines of proteins which have already been pre-phosphorylated at a specific motif recognized by PBDs through the conserved catalytic Ser/Thr kinase domain [13, 16]. PLK1 plays many roles in preparing for and executing mitosis. In particular PLK1 is important for centriole disengagement and maturation [13]. Also, PLK1 has been implicated in contributing to the spindle assembly checkpoint (SAC) by uncoupling Anaphase-Promoting Complex/Cyclosome (APC/C) activation from SAC [17-19]. PLK1 activity itself is also subject to complex regulation, such as phosphorylation by Aurora kinase A, which requires Bora as a co-factor [20-22], and dephosphorylation at Thr210 by the PPP1R12A/MYPT1 phosphatase [23]. One example for the complex regulation of PLK1 activity is that overexpression of Cyclin B2 leads to increased Aurora kinase A activity which causes hyperphosphorylation of PLK1 resulting in accelerated centrosome separation [24]. Confirming its importance for regulating the late phases of the cell cycle, reduced PLK1 expression is often used as an indicator for therapeutic success following drug treatment [25]. Among the substrates of PLK1 are prominent cell cycle-regulating proteins such as CDC25C [16], Cyclin B1 [26, 27], WEE1 [28], LRRK1 [29], KIF20A/MKLP2 [30], KIF2A [31], ECT2 [32], KIZ [33], Protein regulator of cytokinesis 1/PRC1 [34], SGOL1/SGO [35, 36], MISP [37], BORA [38], BUB1B/BUBR1/MAD3L [39], CEP55 [40], FBXO5/EMI1 [18, 41], CENPU/PBIP1 [42], NEDD1 [43], RACGAP1/CYK4 [44, 45], topoisomerase I-binding protein/Topors [46], p73/TP73 [47, 48], TP53BP1 [49], and FOXM1 [50]. Taken together, these substrates exemplify the central role of PLK1 in cell cycle control and oncogenesis [51, 52]. This general importance for PLK1 in cell cycle control is corroborated by the observation that PLK1 is overexpressed in tumors, particularly when p53 function has been compromised [51, 53-55].

The important function of Survivin, CDC25C, and PLK1 is that their expression promotes cell division, which explains their oncogenic potential. Thus, it is important to understand the regulation of their expression, particularly the downregulation of their genes in order to halt the cell cycle.

The transcription factor p53 is a well-studied tumor suppressor and regulates a large number of target genes [56]. Inactivation of p53 leads to the deregulation of several signaling pathways which are important for the development of cancer [57]. Among the target genes of p53 many are downregulated upon p53 activation. Several mechanisms had been suggested for transcriptional repression by p53 [58-60]. Recently, a meta-analysis of genome-wide data sets on gene expression and chromatin immunoprecipitation (ChIP) showed that p53 binding solely correlates with activation of transcription [61].

However, p53-dependent repression of the three key cell cycle genes *Survivin*, *CDC25C*, and *PLK1* had been reported by several groups to be mediated by direct binding of p53 to these targets [62-71]. Furthermore, some reports on the regulation of these genes had proposed models that are entirely or partially conflicting. *CDC25C* was initially even reported to be transcriptionally activated by p53 [72]. Transcriptional repression of *Survivin*, *CDC25C*, and *PLK1* by p53 has been proposed to be mediated by several mechanisms: binding of p53 to a p53 binding site (p53BS) in the three genes [62-64, 67-69], the p53-NF-Y-CCAAT pathway for *CDC25C* [73, 74], a p53-Sp1 pathway for *Survivin* [66, 75], alternate p53-E2F1 pathways for *Survivin* and *PLK1* [62, 70, 76], p21-independent regulation of *CDC25C* by p53 through cell cycle-dependent elements (CDE) and cell cycle genes homology regions (CHR) [63], p21-dependent regulation of *PLK1* and *CDC25C* through CDE/CHR sites [53, 63, 77], a p53-p21-RB/E2F2 pathway for *Survivin* [67], a p53-p21-E2F4 pathway for *Survivin* and *CDC25C* [78], and the p53-p21-DREAM (DP, RB-like, E2F4, and MuvB) pathway for *Survivin* [79]. While most reports on p53-dependent repression of these genes imply direct binding of p53 to the target promoter, a recent meta-analysis suggests that the DREAM complex plays a central role in regulating many genes which are downregulated by p53 [61]. Thus, this study implicated an indirect p53-dependent mechanism of repression without p53 contacting the promoters of repressed genes [59, 61].

DREAM binding to its target DNA is the final step of a pathway that can be initiated by cell stress such as DNA damage. Induction of p21 expression by p53 leads to inhibition of cyclin-dependent kinases, which causes hypophosphorylation of pocket proteins. Thereby the DREAM complex is stabilized which mediates downregulation of its target genes [59, 79-81]. The DREAM complex was shown to bind specifically to CDE and CHR sites that can be found in promoters of genes which are expressed in the late phases of the cell cycle [82-85]. The resulting p53-p21-DREAM-CDE/CHR pathway has been reported to mediate transcriptional repression of *Cyclin B2 (CCNB2)*, *KIF23*, and *PLK4* [59, 83, 86]. These results stand in contrast to several observations reported on the p53-dependent downregulation of *Survivin*, *CDC25C*, and *PLK1*.

Here, we provide evidence that *Survivin*, *CDC25C*, and *PLK1* are not directly repressed by p53. On the contrary, we show that p53-dependent repression employs p21 and the DREAM complex. Differential use of the DREAM-binding CDE and CHR sites mediates repression of these genes.

## RESULTS AND DISCUSSION

### p53-dependent downregulation of *Survivin*, *CDC25C*, and *PLK1* requires p21

**Figure 1:**
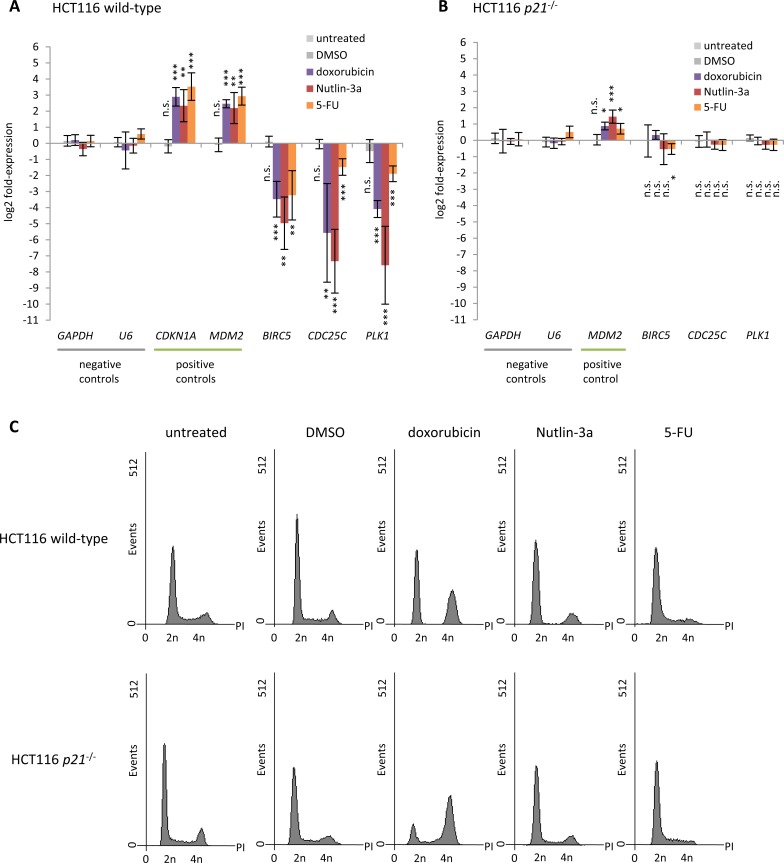
p53-dependent repression of *Survivin* (*BIRC5*), *CDC25C*, and *PLK1* requires p21 Log_2_ fold-change of mRNA expression in HCT116 **A.** wild-type and **B.** *p21*^−/−^ cells treated with doxorubicin, Nutlin-3a, or 5-FU for 24 h normalized to untreated cells. Cells treated with DMSO served as a control. *GAPDH* and *U6* served as negative controls, while *CDKN1A* and *MDM2* were assessed as positive controls. Expression levels were evaluated by comparison to *GAPDH* expression levels using the unpaired Student's t-test. Experiments were performed with two biological replicates and three technical replicates each (*n* = 6). *p ≤ 0.05; **p ≤ 0.01; ***p ≤ 0.001. **C.** Flow cytometry of propidium iodide-stained cells used in **A.** and **B**.

Regulation of *Survivin*, *CDC25C*, and *PLK1* by p53 was tested in HCT116 wild-type cells upon stimulation with the DNA damaging agent doxorubicin, the MDM2 inhibitor Nutlin-3a, or the pyrimidine analogue 5-fluorouracil (5-FU) which activate the p53 pathway. Untreated cells and cells treated with the solvent DMSO served as controls. In agreement with most previous reports [61], we find the expression of *Survivin*, *CDC25C*, and *PLK1* to be downregulated, while the positive controls *CDKN1A* (*p21*) and *MDM2* mRNA are significantly upregulated upon induction of p53 (Figure 1). These results show that downregulation of *Survivin*, *CDC25C*, and *PLK1* is a common event after activation of p53 by various stimuli. Flow cytometry indicates that treatment with the three different drugs causes cell cycle arrest at G_1_/S or G_2_/M transition in a large portion of the cells (Figure 1C). Down-regulation by p53 has been suggested for *Survivin* [78, 79, 87, 88], *CDC25C* [78, 87], and *PLK1* [53, 77, 89] to depend on p21. Therefore, we also tested for mRNA expression of these genes before and after stimulation of the p53 pathway in HCT116 *p21*^−/−^ cells. Importantly, the p53-dependent downregulation of *Survivin*, *CDC25C*, and *PLK1* is essentially lost in HCT116 *p21*^−/−^ cells in contrast to activation of *MDM2* (Figure 1B). When comparing HCT116 *p21*^−/−^ to HCT116 wild-type cells by flow cytometry, profiles indicate a change in cell cycle phase distribution, particularly the increase in the number of cells in S phase, after treatment with doxorubicin and Nutlin-3a (Figure 1C). This observation likely stems from a reduced ability of p21-deficient cells to arrest. This is in agreement with the finding that p21 is required for sustained G_1_/S and G_2_/M arrest [90, 91]. Taken together, these results support indirect repression of *Survivin*, *CDC25C*, and *PLK1* via p21 and question reports of direct regulation by p53.

### Downregulation of *Survivin, CDC25C*, and *PLK1* by p53 is evolutionarily conserved

**Figure 2:**
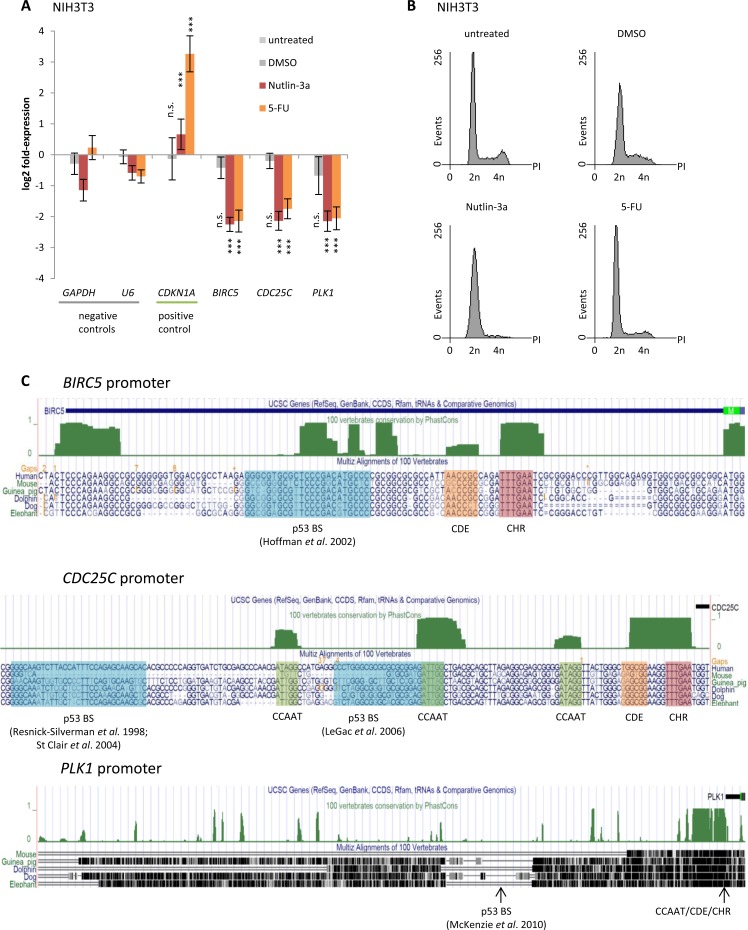
Downregulation of *Survivin* (*BIRC5*), *CDC25C*, and *PLK1* by p53 is evolutionarily conserved between mouse and human **A.** Log_2_ fold-change of mRNA expression in NIH3T3 cells treated with Nutlin-3a or 5-FU for 24 h normalized to untreated cells. Cells treated with DMSO served as a control. *GAPDH* and *U6* served as negative controls, while *CDKN1A* was used as a positive control. Expression levels were evaluated by comparison to *GAPDH* expression levels using the unpaired Student's t-test. Experiments were performed with two biological replicates and three technical replicates each (*n* = 6). *p ≤ 0.05; **p ≤ 0.01; ***p ≤ 0.001. **B.** Flow cytometry analysis of propidium iodide-stained cells used in **A.** and **B.**. **C.** UCSC genome browser graphs [94, 95] displaying segments of the *Survivin (BIRC5)*, *CDC25C*, and *PLK1* promoters. The vertebrate conservation track PhastCons [93] highlights phylogenetic footprints (green) of previously described CCAAT, CDE, and CHR elements, as well as of potential p53 binding sites (p53BS) which were proposed previously and analyzed in this study.

Gene regulation upon induction of p53 is often found to be evolutionarily conserved, as is cell cycle-dependent regulation of these genes [59, 82, 83, 86]. Thus, we tested for p53-dependent regulation of *Survivin*, *CDC25C*, and *PLK1* also in mouse NIH3T3 cells. Indeed we find all three genes to be downregulated upon treatment with Nutlin-3a or 5-FU (Figure 2A). Flow cytometry profiles of these cells show that treatment with 5-FU leads to minor changes in cell cycle distribution compared to cells left untreated or treated with the DMSO solvent. Treatment with Nutlin-3a caused an accumulation in G_1_ phase (Figure 2B).

Notably, DNA sequences important for gene regulation display significant conservation compared to non-functional interspersed DNA [92]. Thus, it is expected that promoter elements mediating p53-dependent regulation are conserved as well. Consequently, we searched for phylogenetic conservation of described regulatory elements in the promoters of *Survivin*, *CDC25C*, and *PLK1* using PhastCons track [93] provided by the UCSC genome browser [94, 95]. Interestingly, the p53 binding sites described for *Survivin* [62], *CDC25C* [63, 64, 72], and *PLK1* [69] display, if at all, only weak phylogenetic footprints (Figure 2C). In contrast, CDE and CHR elements that were reported to be essential for the cell cycle-dependent regulation of *Survivin* [96, 97], *CDC25C* [98-100], and *PLK1* [101] display significant phylogenetic conservation. Considering that p53-dependent repression of *Survivin*, *CDC25C*, and *PLK1* is evolutionarily conserved between mouse and human (Figures 1A and 2A), it is likely that the underlying mechanism including the important promoter elements is conserved as well.

### p53-dependent repression is mediated via CDE/CHR elements but not through CCAAT-boxes or putative p53 binding sites

**Figure 3:**
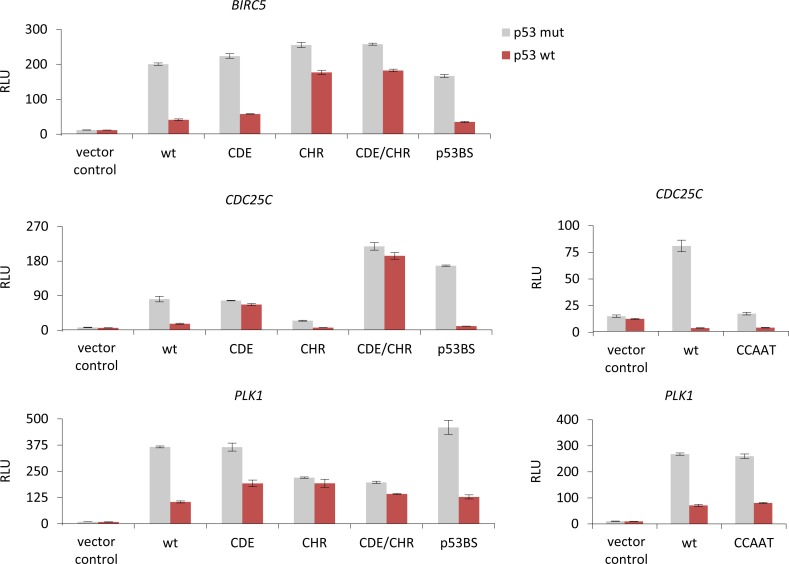
p53-dependent repression of *Survivin* (*BIRC5*), *CDC25C*, and *PLK1* is mediated via CDE/CHR elements but not through CCAAT-boxes or p53 binding sites Luciferase reporter assays from wild-type (wt) or mutant *Survivin*, *CDC25C*, and *PLK1* promoter constructs transfected into HCT116 *p53*^−/−^ cells. Mutants of the potential transcription factor binding sites CDE, CHR, CDE/CHR (CDE and CHR mutated), p53BS, or deletions of CCAAT-boxes were tested. Plasmid constructs were cotransfected in HCT116 *p53*^−/−^ cells with p53R175H mutant (p53 mut) or p53 wild-type (p53 wt) expression vectors. The pGL4.10 luciferase reporter vector was used as negative control. Relative luciferase units (RLU) are shown.

To assess possible elements involved in p53-dependent gene regulation, we created mutants in the potential transcription factor binding sites of target promoters and tested them together with wild-type promoters in luciferase reporter assays for their response to p53 expression. We examined whether CDE/CHR elements or reported putative p53 binding sites are involved in p53-dependent regulation of these promoters. Furthermore, we tested whether *CDC25C* and *PLK1* promoters employ CCAAT-boxes for p53-dependent regulation as suggested by the NF-Y/p53 liaison [53, 73, 74, 102, 103]. Luciferase reporter assays were performed with wild-type (wt) and mutant *Survivin*, *CDC25C*, and *PLK1* promoter reporter constructs after transfection of p53 wild-type (p53 wt) or p53R175H mutant (p53 mut, as a negative control) plasmids (Figure 3). The p53R175H mutant has lost its ability to transactivate genes such as *p21* and does not display a gain-of-function effect on the regulation of the reported DREAM-CDE/CHR target genes *CCNB2*, *KIF23*, and *PLK4* [59, 83, 86]. The activity of wild-type promoters is downregulated by p53 wt similar to the corresponding mRNA (Figure 3). When testing promoter elements necessary for repression, we observed that both the CDE and the CHR sites are required for p53-dependent repression of *Survivin*, *CDC25C*, and *PLK1*. However, the contribution of both elements to p53-dependet repression varies. While *Survivin* and *PLK1* predominantly require the CHR for downregulation upon p53 activation, the *CDC25C* promoter mainly relies on the CDE (Figure 3). Thus, p53-dependent repression of *Survivin* and *PLK1* resembles that of the *CCNB2* and *KIF23* promoters [59, 86], whereas *CDC25C* displays CDE/CHR-mediated downregulation similar to the *PLK4* promoter [83].

Other transcription factor binding sites implicated in p53-dependent repression are CCAAT-boxes which function as activating elements after binding NF-Y [104]. Here we show that deletion of the three CCAAT-boxes in the *CDC25C* promoter yielded a substantially lower activity compared to the wild-type construct in reporter assays. Importantly, the low reporter activity of the deletion mutant was further repressed upon expression of wild-type p53 (Figure 3). The difficulty to further repress CCAAT-box deletion mutants by p53 led us and others earlier to the false interpretation that CCAAT-boxes are required for p53-dependent transcriptional repression [73, 74]. Mutation of the CCAAT-box in *PLK1* and deletion of the CCAAT-boxes in *CDC25C* do not significantly change p53-dependent downregulation (Figure 3). Concordantly, in a meta-analysis we showed that CCAAT-boxes do not correlate with p53-dependent repression independently of pocket protein complexes such as DREAM [61].

Importantly, the p53 consensus sites proposed for *Survivin* [62], *CDC25C* [63, 64], and *PLK1* [69] do not appear to be functional as they do not significantly alter p53-dependent repression of these promoters (Figure 3).

Focusing on the *CDC25C* gene, it becomes evident that the history of p53 site descriptions in its promoter and p53-dependent regulation is long and contradictory. A site within the human *CDC25C* promoter, closely related to the p53 consensus, was originally reported to weekly bind p53 in electrophoretic mobility shift assays and, when placed into a heterologous reporter system with an adenovirus *E1b* minimal promoter, to activate p53-dependent transcription [72]. However, we showed that human *CDC25C* transcription is downregulated by p53 and the p53 consensus site proposed earlier is not involved in this regulation [73]. Moreover, the proposed p53 element is not evolutionary conserved and absent in the mouse promoter (Figure 2C). In agreement with this observation, we confirmed that this promoter is nonetheless downregulated by p53 [99]. Furthermore, when this p53 consensus site is mutated in reporter assays using the human *CDC25C* gene, p53-dependent transcriptional repression is still observed, strongly arguing against a role of this site in p53-dependent repression (Figure 3). Later, an indirect mechanism for repression requiring p21 was described [87]. In contrast to these reports which exclude a role for the p53 consensus element in p53-depedent regulation, the group originally describing activation through this p53 consensus site later suggested it to be important for repression by p53. The proposed mechanism involved direct binding and repression by p53 through this element if eight GC-rich base pairs are present upstream of the p53 consensus element [63]. In a complex model involving two promoter regions for regulation of *CDC25C*, this report also implicated CDE/CHR sites in p53-dependent regulation, but excluded p21 to be essential [63]. In a recent study, it was suggested that p21 is required for p53-depedent transcriptional repression of *CDC25C*. With regard to p53 sites in the target promoter, the report discusses these elements as ‘nonfunctional sites’ [78].

**Figure 4:**
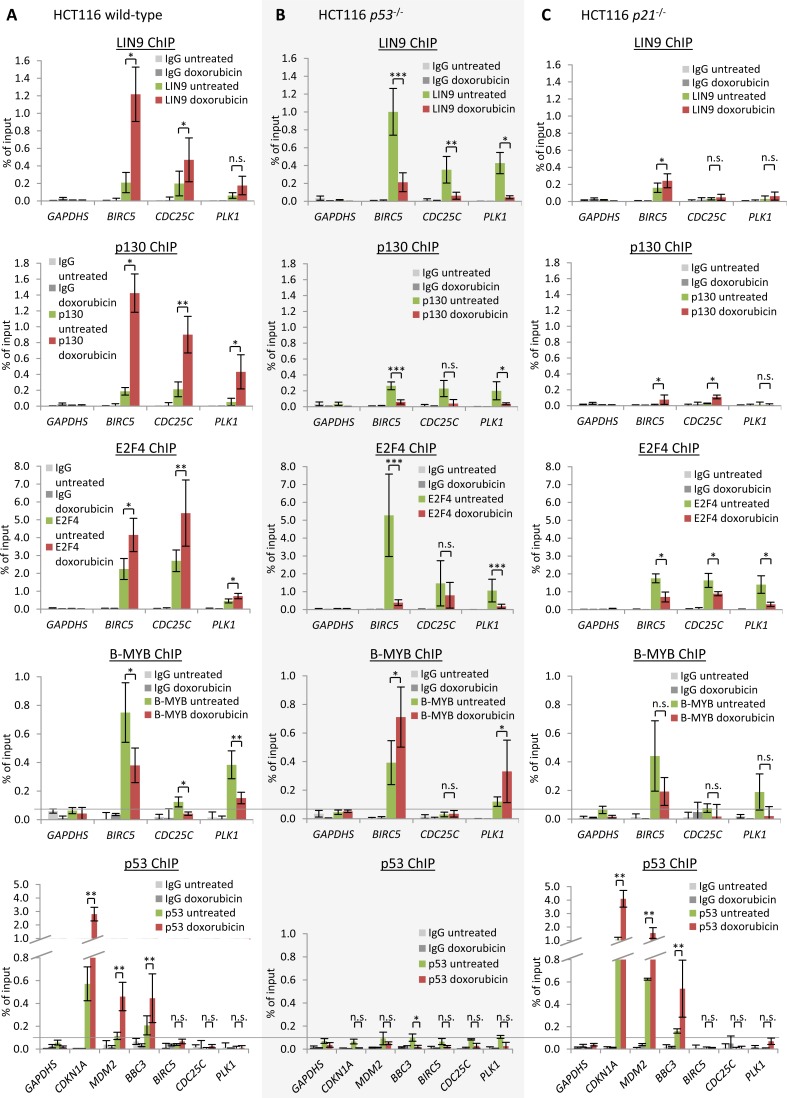
DREAM binding to *Survivin* (*BIRC5*), *CDC25C*, and *PLK1* is increased upon DNA damage induction and depends on p21 Protein binding to the *Survivin*, *CDC25C*, and *PLK1* promoters in untreated or HCT116 cells treated by doxorubicin for 48 h: HCT116 **A.** wild-type, **B.** *p53*^−/−^, or **C.** *p21*^−/−^ cells. Binding of protein was tested by chromatin immunoprecipitation followed by real-time PCR. Protein binding to the GAPDHS promoter served as a negative control. One representative experiment with three technical replicates (*n* = 3) is displayed. Significance was tested using the paired Student's *t*-test; n.s. not significant; *p ≤ 0.05; **p ≤ 0.01; ***p ≤ 0.001.

Discussion on p53 sites in the *CDC25C* promoter became even more complex when, in addition to the distal site debated above [63, 72, 73, 99], Le Gac and coworkers described a proximal p53 consensus site. They suggested that this element recruits p53 as well as DNMT1 and HDAC1, resulting in DNA methylation and thus silencing of the *CDC25C* gene [64]. This second proposed p53 consensus site is also not phylogenetically conserved (Figure 2C). A functional p53 consensus element would require binding of p53 to the gene. However, p53 binding to the *CDC25C* promoter as shown by ChIP is not above background levels and does not increase upon DNA damage (Figure 4).

Comparing levels of p53 binding observed for positive (e. g. *CDKN1A* and *MDM2*) and negative (e. g. *GAPDHS*) controls, p53 binding to *CDC25C* appears to be at background level (Figure 4). Consistent with this result are observations from genome-wide ChIP studies, as none of these reports found significant binding above background of p53 to the *CDC25C* gene [88, 105-108]. These results also imply that putative p53 consensus sites in the promoter in fact do not bind p53 protein.

Also for the regulation of *PLK1* conflicting observations have been reported [51]. While Zhu and coworkers had implicated the CDE/CHR site in mediating p21-dependent repression of *PLK1*, McKenzie *et al.* suggested a p53 consensus element to be responsible for direct repression by recruiting p53 to the target promoter [69, 77]. Similarly to *CDC25C* and *PLK1*, there are also conflicting reports on the importance of a p53 consensus site in the *Survivin* promoter. While one study proposed direct repression by p53 via a p53 consensus element [62], Löhr *et al.* found this p53 site to be dispensable for regulation of *Survivin* and favored an indirect p21-dependent repression mechanism [87]. With regard to results from ChIP experiments, not one of six genome-wide studies found significant binding of p53 to *Survivin* or *PLK1* [88, 105-109]. Thus, these six publications made observations which are consistent with the results presented and discussed here (Figure 4). Notably, there is no evidence for phylogenetic conservation of the putative p53 elements in any of the three genes discussed (Figure 2C). This is consistent with the lack of significant p53 binding above background to *CDC25C*, *PLK1,* or *Survivin* after induction of DNA damage (Figure 4). Also testing the putative p53 consensus element in *Survivin* promoter regulation by reporter assays did not confirm any role of this region in p53-dependent transcriptional repression (Figure 3).

In summary, we conclude that the phylogenetically conserved CDE and CHR elements mediate p53-dependent repression of *Survivin*, *CDC25C*, and *PLK1*, while CCAAT-boxes as well as the proposed p53 consensus sites are not involved.

### DREAM binding is increased upon induction of DNA damage and depends on p21

Protein binding to the cell cycle genes was tested by ChIP assays. We used chromatin from HCT116 cells before or after induction of DNA damage by doxorubicin. The promoters of *Survivin*, *CDC25C*, and *PLK1* bind p130 and E2F4, two representative components of the repressive DREAM complex. Binding of p130 and E2F4 to the promoters is significantly increased after doxorubicin-induced DNA damage (Figure 4A).

As potential promoter binding sites for DREAM in cells, we had presented results from ChIP experiments with promoter transgenes showing that E2F4, LIN9, and p130 binding is lost when CHR elements are mutated [59]. Furthermore, several *in vitro* binding studies revealed that B-MYB and FOXM1 require CHR sites, and DREAM components require CDE and CHR sites for binding [82, 83, 85, 86, 110]. Considering these reports and results presented here (Figures 3 and 4), the data suggest DREAM binding to CDE/CHR sites in the promoters of *CDC25C*, *PLK1,* and *Survivin* genes also *in vivo*.

Consistent with a DREAM- or MuvB-based transcriptional mechanism is also the observation that LIN9, a shared component of DREAM and MuvB, binds the *Survivin*, *CDC25C*, and *PLK1* genes (Figure 4A). It has been shown that *Lin9* is required for the regulation of *Survivin* and *PLK1* genes [111]. Furthermore, *Plk1* is deregulated in mouse embryonic fibroblasts in which functional parts of *Lin9* have been deleted [112]. Moreover, *Survivin* was previously shown to recruit DREAM upon induction of p53 [79]. Doxorubicin-induced DREAM binding was also observed at the DREAM-CDE/CHR target promoters of *CCNB2, KIF23,* and *PLK4* [59, 83, 86].

In contrast to DREAM binding, binding of the MMB component B-MYB was reduced at *Survivin*, *CDC25C*, and *PLK1* promoters after doxorubicin treatment, while binding of the MuvB core component LIN9 was found to be enriched at the *Survivin* and *CDC25C* promoters, but not at the *PLK1* promoter (Figure 4A). Concordantly, we had observed previously that LIN9 binding is enriched at the *PLK4* promoter after doxorubicin treatment, but not at the promoters of *CCNB2* and *KIF23* [59, 83, 86]. These variations in the LIN9 binding pattern may result from different affinities of DREAM and MMB to the different promoters. Notably, *Survivin* and *PLK1* were previously reported to bind the DREAM complex [79, 113, 114] and in a genome-wide screen of quiescent T98G cells *Survivin, CDC25C,* and *PLK1* were listed as potential DREAM targets [115].

When looking at p53 association with DNA by ChIP, no significant binding of p53 was observed to the promoters of *Survivin, CDC25C,* and *PLK1*, in contrast to the promoters of cyclin-dependent kinase (CDK) inhibitor *p21* (*CDKN1A*), *MDM2,* and *PUMA* (*BBC3*) which served as positive controls (Figure 4A). These results indicate indirect repression of *Survivin*, *CDC25C*, and *PLK1*. Previous studies showed that transcription of *p21* is induced by p53 [116]. Recent evidence from a knockout mouse model suggests that p21 is required for p53-dependent repression of *Plk1* expression [89]. We find that p21 is essential for p53-dependent repression of *Survivin, CDC25C,* and *PLK1* (Figure 1). Inactivation of CDKs by p21 causes hypophosphorylation of p130 [59, 79]. As a consequence, the DREAM complex forms by switching binding on the MuvB core from B-MYB to binding of p130 and E2F4/DP1. During this shift from MMB to DREAM, the MuvB core complex was suggested to remain bound to CHR sites in the target promoters [59, 82]. In summary, activation of p53 can cause a switch on the MuvB core from the activating MMB to the repressive DREAM complex [59, 79, 80, 82]. Thus, we tested whether binding of DREAM to the *Survivin*, *CDC25C*, and *PLK1* genes depends on p53 and p21. In contrast to wild-type HCT116 cells, ChIP assays from HCT116 *p53*^−/−^ cells reveal a decreased binding of the DREAM components p130 and E2F4 after doxorubicin treatment (Figure 4B). In HCT116 *p21*^−/−^ cells, we observed generally low levels of DREAM binding to the target genes and also no increase after doxorubicin-induced DNA damage (Figure 4C). Moreover, we found that E2F4 binding is reduced in HCT116 *p21*^−/−^ cells treated with doxorubicin compared to cells left untreated. Also, in HCT116 *p53*^−/−^ cells no binding of p53 was observed at any promoter, confirming the deficiency in p53 and the specificity of the p53 antibody (Figure 4B). ChIP assays from HCT116 *p21*^−/−^ cells show binding of p53 at *CDKN1A*, *MDM2*, and *BBC3* which served as positive controls, but not at the promoters of *Survivin*, *CDC25C*, and *PLK1* (Figure 4C). These results support the findings from HCT116 wild-type cells which show that p53 does not bind to *Survivin*, *CDC25C*, and *PLK1*.

Taken together, our results suggest that p53-dependent downregulation of *Survivin*, *CDC25C*, and *PLK1* upon doxorubicin-induced DNA damage requires the CDE/CHR elements and a p53- and p21-dependent shift from MMB to DREAM complexes binding to the promoters.

### The transcription factor p53 is not a direct repressor of transcription

It was reported by several groups that *Survivin* [62, 65-68], *CDC25C* [63, 64], and *PLK1* [69, 70] are directly repressed by p53 binding to their promoters. However, it was demonstrated recently that p53 does not act as a repressor, but solely is an activator of transcription [61]. In agreement with this model, we find no evidence for direct repression of *Survivin*, *CDC25C*, and *PLK1* by p53. Four observations lead to this conclusion. First, we find p53-dependent repression of *Survivin*, *CDC25C*, and *PLK1* to be essentially lost in HCT116 cells lacking p21 compared to wild-type cells (Figure 1). Second, the proposed p53 binding sites are not found to be phylogenetically conserved, in contrast to p53-dependent repression of these genes (Figure 2). Third, mutation of proposed p53 binding sites essentially does not impair p53-dependent repression of *Survivin*, *CDC25C*, and *PLK1* promoters (Figure 3). Fourth, binding of p53 to the promoters is not above background in ChIP assays (Figure 4). Therefore, we conclude that direct repression by p53 is not a part of *Survivin*, *CDC25C*, and *PLK1* regulation, supporting the model that direct transcriptional repression is not a function of p53 [61].

### p53-dependent gene repression by the p53-p21-DREAM-CDE/CHR pathway

In general, many other observations made regarding the regulation of *Survivin*, *CDC25C*, and *PLK1* are in accordance with the p53-p21-DREAM-CDE/CHR pathway. For instance, *Survivin* was shown to be downregulated by TGF-β requiring E2F4 and the CDE/CHR element [117]. Since p21 is a known downstream mediator of the TGF-β signaling pathway [118], this finding supports the notion that *Survivin* is repressed through the p53-p21-DREAM-CDE/CHR pathway. Moreover, *Survivin* was shown to be activated by expression of Myc [119]. This finding is also in agreement with the p53-p21-DREAM-CDE/CHR pathway repressing *Survivin*, since Myc was shown to repress the *p21* promoter [120].

More importantly, p53-dependent repression of the *Survivin*, *Cdc25C*, and *Plk1* mouse orthologs was shown to depend on the pocket proteins p107 and p130 [121], which were later identified as components of the DREAM complex [113, 115, 122]. Together with the fact that the CDE and CHR sites are conserved between mouse and human (Figure 2), these observations support the notion that *Survivin*, *CDC25C*, and *PLK1* are targets of the evolutionarily conserved p53-p21-DREAM-CDE/CHR pathway.

### Feedback regulation by p53 targets

Reported results indicate that an autoregulatory feedback loop of PLK1 and p53-related proteins exists. PLK1 can phosphorylate Topors, a ubiquitin and SUMO-1 E3 ligase with p53 as a substrate [46]. Phosphorylation by PLK1 leads to inhibition of Topors' sumoylation activity, but to an enhancement of its ubiquitination activity. Thus, p53 protein levels are reduced after PLK1-dependent phosphorylation of Topors and ubiquitination of p53 [46]. As PLK1 expression is itself negatively regulated by p53, this modulation of p53 degradation through PLK1 activity constitutes a positive autoregulatory feedback loop.

Interestingly, the p53-related family of p73 proteins is also a substrate for PLK1 [47, 48]. PLK1 phosphorylates the TAp73 variants and thereby leads to a reduction of their stability and lowers transcriptional activity of p73 [47, 48]. Some isoforms of p73 and p63 are able to transcriptionally activate *p21* and contribute to cell cycle arrest and induction of apoptosis [123-125]. Therefore, the corresponding positive autoregulatory feedback loop discussed for p53 may apply, more directly but similarly, also for p73 and even p63.

### p53 arrests the cell cycle by coordinated downregulation of many genes

Genes for PLK1 substrates as well as *PLK1* itself appear to be downregulated by the DREAM/CHR pathway [61, 85, 126], e. g. *Cyclin B1/CCNB1* [26, 27], *CDC25C* [16], *WEE1* [28], *Protein regulator of cytokinesis 1/PRC1* [34], *SGOL1/SGO* [35, 36], *BORA* [38], *BUB1B/BUBR1/MAD3L* [39], *CEP55* [40], *FBXO5/EMI1* [18, 41], and *FOXM1* [50, 61, 85]. These genes are expressed during the late cell cycle and were identified among others to bind DREAM and to harbor CHR elements in their promoters [85].

Substrates and interaction partners of Survivin, CDC25C, and PLK1 form a large network which in its complexity is responsible for fine-tuning regulation of the late cell cycle [126]. One example for interdependence of the three factors is the phosphorylation of CDC25C by PLK1, which is responsible for translocation of CDC25C into the nucleus during prophase [127]. In the nucleus, CDC25C activates the Cyclin B/CDK1 complex to drive the cell through mitosis [4]. Of note, most genes participating in the circuitry, e. g. *PLK1, CDC25C, Cyclin B1, Cyclin B2,* and *CDK1/CDC2*, are transcriptionally repressed through CDE/CHR elements [84, 85, 98, 98, 99, 101, 128]. As shown for *Cyclin B2 (CCNB2)*, *KIF23*, *PLK4*, *Survivin, CDC25C*, and *PLK1*, the mechanism of transcriptional repression by p53 appears to be based on the p53-p21-DREAM-CDE/CHR pathway (Figures 1, 2, 3, 4) [59, 83, 86].

Furthermore, p53-dependent repression of *Survivin*, *CDC25C* and many other factors controlling cell division appears to serve the same purpose [61]. Downregulation of genes required for cell cycle progression is a central mechanism by which p53 arrests the cell cycle. In general, most genes downregulated by p53 support cell cycle progression and promote tumorigenesis. Consistently, also *Survivin*, *CDC25C,* and *PLK1* were shown to be overexpressed in tumors [1-3, 6-9, 51, 53-55].

In summary, our data resolve contradictions from earlier reports and support the model that p53 does not repress transcription through direct binding to its target genes. We provide evidence that the key cell cycle genes *Survivin*, *CDC25C*, and *PLK1* are regulated by the p53-p21-DREAM-CDE/CHR pathway. In general, the regulatory network controlled by this pathway leads to an amplification of signals inhibiting cell division upon p53 activation. Cell cycle arrest is achieved through coordinated downregulation of genes which support cell cycle progression. Thus, the p53-p21-DREAM-CDE/CHR pathway appears to constitute an important mechanism for p53 to prevent the development of cancer.

## MATERIALS AND METHODS

### Cell culture and drug treatment

HCT116 wild-type, HCT116 p53−/− and HCT116 p21−/− cells, kindly provided by Bert Vogelstein [90, 91], were grown in Dulbecco's modified Eagle's medium (DMEM; Lonza, Basel, Switzerland) supplemented with 10% fetal calf serum (FCS) (Biochrom, Berlin, Germany) and penicillin/streptomycin and maintained at 37°C and 10% CO_2_. HCT116 cells were treated with doxorubicin (0.2 μg / ml), Nutlin-3a (10 μM), or 5-FU (25 μg / ml) for 24 h. Control cells were treated with the solvent DMSO or left untreated.

### RNA extraction, reverse transcription and semi-quantitative real-time PCR

Total RNA was isolated from cell lines using TRIzol Reagent (Invitrogen) following the manufacturer's protocol. One-step reverse transcription and quantitative real-time PCR were performed with an ABI 7300 Real-Time PCR System (Applied Biosystems, Forster City, CA, USA) using QuantiTect SYBRGreen PCR Kit (Qiagen, Hilden, Germany). Primer sequences can be obtained upon request.

### Flow cytometry

Cells were fixed for at least 12 h at 4°C in one volume phosphate buffered saline/1 mM EDTA and three volumes of absolute ethanol. DNA was stained with propidium iodide at a final concentration of 10 μg/ml in presence of RNase A (10 μg/ml). DNA content per cell was measured by flow cytometry on an LSR II instrument (Becton Dickinson, Franklin Lakes, NJ, USA). Cell sorting was carried out on a FACSVantage SE (Becton Dickinson). Data analysis was carried out with WinMDI 2.9 software.

### Plasmids, transfections, and luciferase assays

The human *Survivin* (*BIRC5*) promoter with a size of 389 bp (nt −205 to +184 from the transcriptional start site, TSS) and the human *PLK1* promoter with a size of 1089 bp (nt −1019 to +70) were amplified from genomic DNA and cloned into the pGL4.10 basic firefly luciferase reporter vector (Promega, Mannheim, Germany). The human *CDC25C* promoter with a size of 1435 bp (nt −1434 to +1) was subcloned into the pGL4.10 basic vector from the previously published pGL3 plasmids [73, 100]. Mutations were introduced with the QuikChange site-directed mutagenesis kit (Agilent Technologies, Santa Clara, CA, USA). Primer sequences used for cloning and creating mutations can be obtained upon request. The human p53 expression plasmids pcDNA-p53wt and pcDNA-p53mut R175H were described previously [86]. Luciferase reporter assays to determine p53-dependent promoter activity were carried out as described previously [59].

### Chromatin immunoprecipitation

ChIP was performed as described previously [59, 83]. Primer sequences can be obtained upon request.
